# Supplementary material for: Contribution of Attenuation of TNF-α and NF-κB in the Anti-Epileptic, Anti-Apoptotic and Neuroprotective Potential of Rosa webbiana Fruit and Its Chitosan Encapsulation
Source: Molecules. 2021 Apr 17;26(8):2347. doi: 10.3390/molecules26082347 (PMC8073239; doi:10.3390/molecules26082347)
Supplement: Supplementary file 1 [file molecules-26-02347-s001.zip › molecules-1166482-supplementary.pdf]

Supplementary Materials

Contribution of Attenuation of TNF-α and NF-κB in the Anti-epileptic, Anti-apoptotic and Neuroprotective Potential of *Rosa webbiana* Fruit and its Chitosan Encapsulation

Anum Firdous [1], Sadia Sarwar [1], Fawad Ali Shah [3], Sobia Tabasum [2], Alam Zeb [4], Humaira Nadeem [5], Abir Alamro [6], Amani Ahmed Alghamdi [6], Arooj Mohsin Alvi [3], Komal Naeem [3] and Muhammad Sohaib Khalid [1]

[1] Departament of Pharmacognosy, Riphah Institute of Pharmaceutical Sciences, Faculty of Pharmaceutical Sciences, Riphah International University, Islamabad 44000, Pakistan
[2] Departament of Biological Sciences, International Islamic University, Islamabad 44000, Pakistan
[3] Departament of Pharmacology, Riphah Institute of Pharmaceutical Sciences, Faculty of Pharmaceutical Sciences, Riphah International University, Islamabad 44000, Pakistan
[4] Departament of Pharmaceutics, Riphah Institute of Pharmaceutical Sciences, Faculty of Pharmaceutical Sciences, Riphah International University, Islamabad 44000, Pakistan
[5] Departament of Pharmaceutical Chemistry, Riphah Institute of Pharmaceutical Sciences, Faculty of Pharmaceutical Sciences, Riphah International University, Islamabad 44000, Pakistan
[6] Departament of Biochemistry, College of Science, King Saud University, PO Box 22452, Riyadh 11495, Saudi Arabia

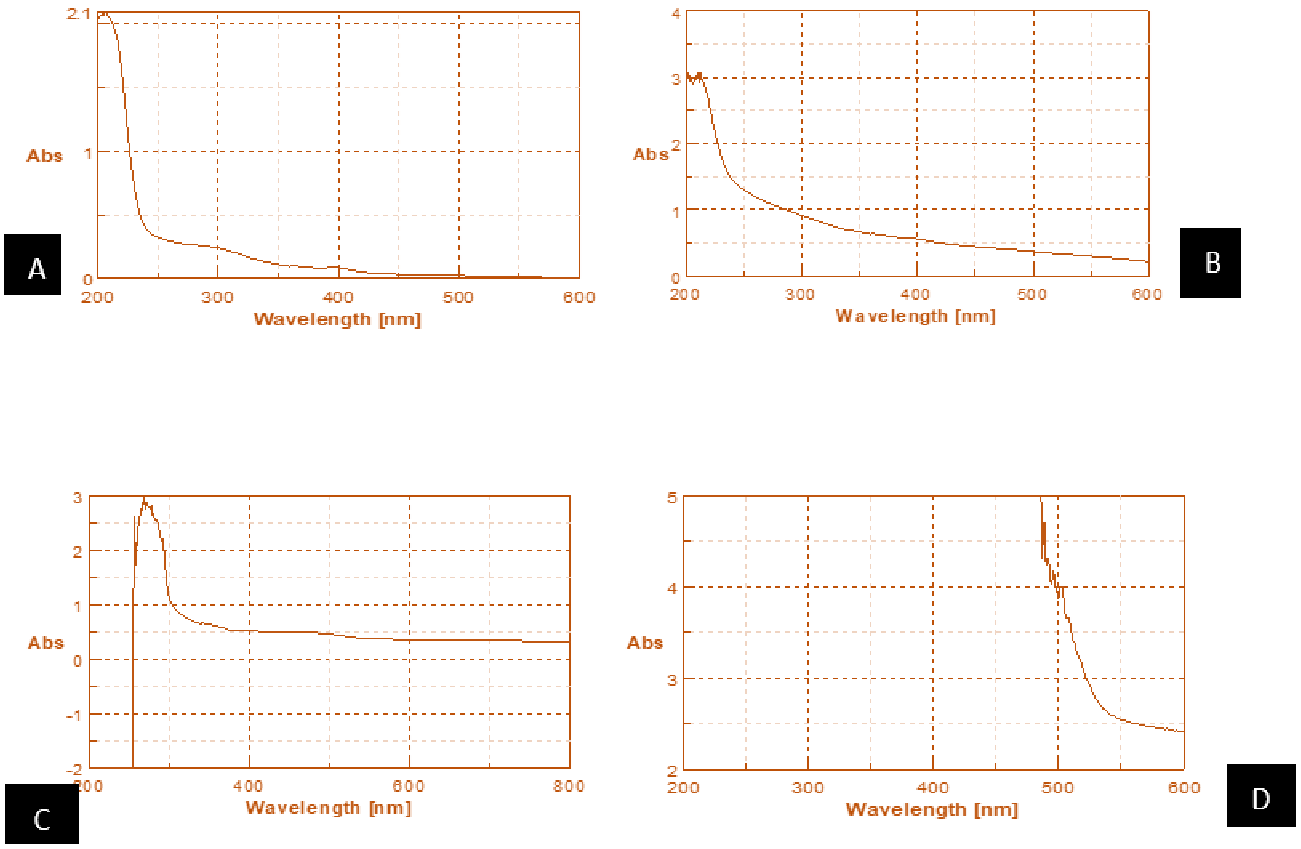

Figure S1. (**A**) CS solution absorbance peak at 210 nm. (**B**) CS-TPP NP's absorbance peaks 210 and 212 nm. (**C**) P.E UV spectrum. (**D**) P.E NP's UV spectrum.
